# Genome-Wide Identification and Functional Analysis of the Roles of *SAUR* Gene Family Members in the Promotion of Cucumber Root Expansion

**DOI:** 10.3390/ijms24065940

**Published:** 2023-03-21

**Authors:** Jie Luan, Ming Xin, Zhiwei Qin

**Affiliations:** Key Laboratory of Biology and Genetic Improvement of Horticultural Crops (Northeast Region), Ministry of Agriculture, College of Horticulture and Landscape Architecture, Northeast Agricultural University, Harbin 150030, China

**Keywords:** cucumber, auxin, small auxin-up RNA, *CsSAUR31*, development

## Abstract

Auxin serves as an essential regulator of the expression of many different genes in plants, thereby regulating growth and development. The specific functional roles of members of the SAUR (small auxin-up RNA) auxin early response gene family in the development of cucumber plants, however, remain to be fully clarified. Here, 62 SAUR family genes were identified, followed by their classification into 7 groups that included several functionally associated cis-regulatory elements. Phylogenetic tree and chromosomal location-based analyses revealed a high degree of homology between two cucumber gene clusters and other plants in the Cucurbitaceae family. These findings, together with the results of an RNA-seq analysis, revealed high levels of *CsSAUR31* expression within the root and male flower tissues. Plants overexpressing *CsSAUR31* exhibited longer roots and hypocotyls. Together, these results provide a basis for further efforts to explore the roles that *SAUR* genes play in cucumber plants, while also expanding the pool of available genetic resources to guide research focused on plant growth and development.

## 1. Introduction

Auxin is a plant hormone that regulates the division, differentiation, and patterning of cells, thereby influencing virtually every aspect of the growth and development of higher plants [1,2]. Genes that exhibit early responses to auxin exposure include members of the *Aux/IAA*, *GH3*, and small auxin-up RNA (*SAUR*) gene families [3], of which the *SAUR* genes are the most abundant. The advent and proliferation of genome sequencing technologies have led to the production of large volumes of data, leading to the identification and characterization of the *SAUR* gene family in many plants. For example, *Arabidopsis* plants have been found to express 79 *SAUR* genes [3], while 56, 71, 134, 99, 65, 66, 72, and 68 members of these gene family have been characterized in rice, sorghum, potato, tomato, watermelon, melon, foxtail millet, and longan plants, respectively [4,5,6,7,8,9,10]. In general, the *SAUR* family includes 60–140 genes in most plant species, with the majority of these genes being a single exon in length. In addition to their short lengths, the half-lives of SAUR mRNAs and proteins tend to be quite short within plant cells [11].

Recent studies have conducted functional characterization of specific *SAUR* genes in both rice and *Arabidopsis* plants. In *Arabidopsis*, for example, *SAUR63* can function as a promoter of organ elongation [12,13,14], while *SAUR19* positively regulates cellular expansion [15]. *SAUR26* subfamily genes play an important role in the thermal response of the growth structure among natural accessions of *Arabidopsis thaliana* [16], and *SAUR41* controls gravitropic root growth and the expansion of root cells [17]. Mechanistically, these SAUR proteins can act as hubs for the integration of signals from both the environment and plant hormonal inputs, thereby coordinating plant development [18]. *AtSAUR32* plays an important role in drought stress adaptation through ABA signal transduction [19]. Members of the *SAUR41* subfamily can regulate plant sensitivity to abscisic acid (ABA), with the overexpression and quadruple mutation of *SAUR41* resulting in extensive root ring and loose root ring phenotypes, respectively [20]. *SAUR17* and *SAUR50* play opposing roles in the regulation of the activity of PP2C-D1, with light-mediated signaling reducing *SAUR17* activity while promoting the upregulation of *SAUR50* and other *SAUR* genes on the inner side of the hook and cotyledon cells, which influences the development of cotyledons and root tip hooks [21]. The SAUR15/BRI1/PM H+- ATPase brassinosteroid (BR) signal transduction pathway can promote the development of many plant organs by driving cellular expansion [22]. When *SAUR53* is ectopically overexpressed, it can lead to cell and organ elongation and to impaired apical hook development, although *SAUR53*, in contrast to most members of this gene family, is not auxin-inducible [23]. The OsSAUR45 protein in rice can inhibit *OsPIN* and *OsYUCCA* gene expression, thereby modulating the synthesis and transport of auxin [24]. The expression of a range of *SAUR* genes including *SAUR*5, 7, 11, 17, 33, 36–48, and 57 was shown to be associated with exogenous auxin application timing [25]. In tomato plants, *SlSAUR69* overexpression can reduce polar auxin transport, while enhancing sensitivity to ethylene in a manner that drives tomatoes to begin ripening prematurely [26]. Wheat *TaSAUR75* overexpression in *Arabidopsis* also improves the ability of these plants to tolerate salt and drought stress conditions [27].

Cucumbers (*Cucumis sativus* L.) are widely cultivated and among the most economically valuable members of the Cucurbitaceae family, in addition to serving as a common model for studies of plant fruit ripening and sex determination. In the present study, a bioinformatics approach was used to analyze members of the cucumber *SAUR* gene family with a focus on gene structures, conserved motifs, the chromosomal locations of these genes, their phylogenetic relationships, and any cis-acting promoter elements associated therewith. In addition, an in-depth analysis of the role that *SAUR31* plays as a regulator of the growth and development of cucumber plants was conducted by overexpressing this gene in these plants. Together, these results offer comprehensive, genome-wide insights into the composition of the *SAUR* gene family in cucumbers, while providing promising genetic resources for future breeding efforts.

## 2. Results

### 2.1. CsSAUR Gene Identification and Phylogenetic Analysis

An initial BLAST search of the cucumber genome database was performed using SAUR family protein sequences from melon, watermelon, and *Arabidopsis*. This whole-genome search ultimately led to the identification of 62 *SAURs* that were named *CsSAUR1*–*CsSAUR62*. These *CsSAUR* family members ranged in molecular weight values from 9.474 kDa (*CsSAUR47*) to 86.253 kDa (*CsSAUR37*) (Table S2) and exhibited predicted pI values from 4.77 (*CsSAUR40*) to 10.38 (*CsSAUR26*). The encoded proteins ranged from 84 (*CsSAUR47*) to 746 (*CsSAUR37*) amino acids in length, largely in line with predicted molecular weight values, and had instability indices from 21.94 (*CsSAUR5*) to 87.94 (*CsSAUR60*). The most stable members of this gene family were *CsSAUR6*, *CsSAUR58*, *CsSAUR54*, *CsSAUR34*, *CsSAUR26*, *CsSAUR7*, *CsSAUR57*, and *CsSAUR5*, as they exhibited instability coefficient values of less than 40. Average hydrophobicity values were between −0.962 (*CsSAUR27*) and 0.054 (*CsSAUR20*), consistent with the hydrophilicity of the encoded proteins.

**Figure 1:**
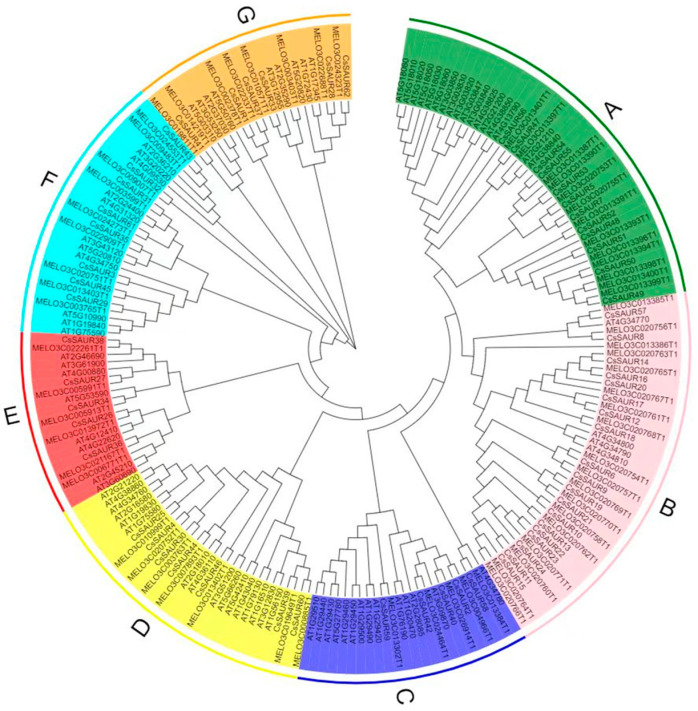
Phylogenetic tree incorporating SAUR proteins from cucumber, melon, and *Arabidopsis thaliana.* The background colors on the outer ring highlight different cluster branches.

A neighbor-joining method was used to assess the phylogenetic relationships among these *CsSAURs*, by constructing an unrooted phylogenetic tree based on the full-length protein sequences for these 62 CsSAURs, as well as 79 *Arabidopsis* SAURs and 66 melon SAURs, with 1000 bootstrap replicates (Figure 1). These proteins were classified into seven groups (A–G), with groups of different proteins from all three species included in clusters B, E, F, and G, and most cucumber and melon SAURs were present in cluster B. Cluster A contained CsSAURs from chromosome 7, and cluster B contained CsSAURs from chromosome 2.

### 2.2. CsSAUR Gene Chromosomal Distributions

**Figure 2:**
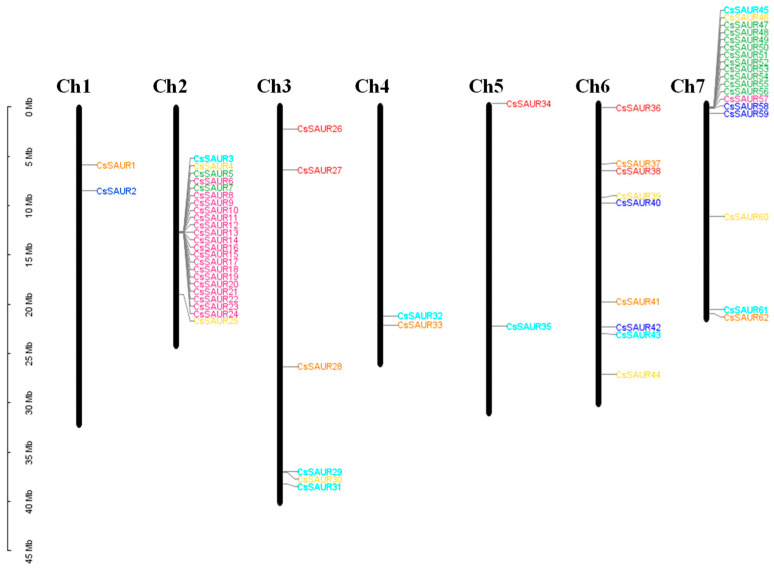
The chromosomal locations of *CsSAUR* genes. Members of the same group are presented in the same color. The provided scale is in centimorgans (Mb).

To further study the evolution of these *CsSAUR* genes, the chromosomal locations of the 62 identified gene family members were assessed, revealing them to be distributed on all 7 chromosomes (Figure 2). This included 23 members on chromosome 2, while chromosomes 1, 4, and 5 contained the fewest, with only 2 members. A cluster of genes including *CsSAUR3*–*CsSAUR25* was present in the middle of chromosome 2, while *CsSAUR45*–*CsSAUR59* were clustered in the upper arm of chromosome 7, and other gene family members were spread throughout the remainder of the genome.

### 2.3. Analyses of CsSAUR Gene Structures and Conserved Motifs

**Figure 3:**
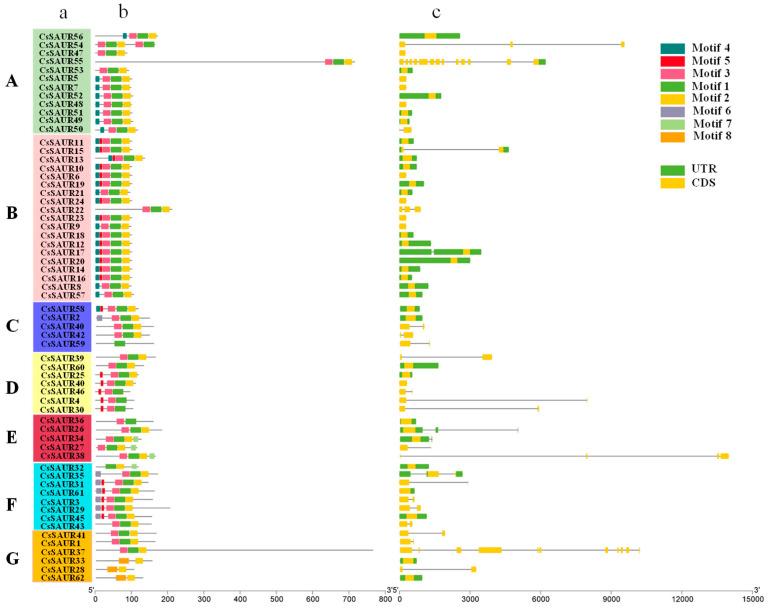
CsSAUR motifs and gene structures. (**a**) Clustering analysis. (**b**) Motif architecture. (**c**) CDS and UTR. The background colors and the letters highlight different cluster branches.

To explore the conserved motif distributions in the identified *CsSAUR* genes, TBtools was used, leading to the identification of eight conserved motifs, for which the positional information and logos are provided in Figure 3b. The majority of these CsSAUR proteins harbored three motifs (motifs 1–3), suggesting that these domains may be important regulators of cucumber development. An additional motif (motif 4) was also present in 27 of these CsSAURs, located in two gene clusters on chromosomes 2 and 7, suggesting that these CsSAURs may exhibit distinct functional characteristics. CsSAUR59 harbored only a single motif, motif 1, while four CsSAURs harbored two motifs, including CsSAUR28, CsSAUR 33 and CsSAUR62 (motif 2 and motif 8), and CsSAUR 36 (motif 1 and motif 3). Of these motifs, motifs 1 and 2 were the most widely distributed, suggesting that they are particularly critical to the growth and development of cucumbers.

To explore *CsSAUR* structural evolution, the intron and exon organization were assessed with TBtools (Figure 3c). Those genes in the same group typically exhibited similar numbers of introns, and the majority of these *CsSAUR* genes were found to only contain exons. The low numbers of intron-containing genes in this family may be attributable to the loss of introns over the course of evolution or the acquisition of introns in a select subset of *CsSAURs.*

### 2.4. Identification of Cis-Acting Elements

**Figure 4:**
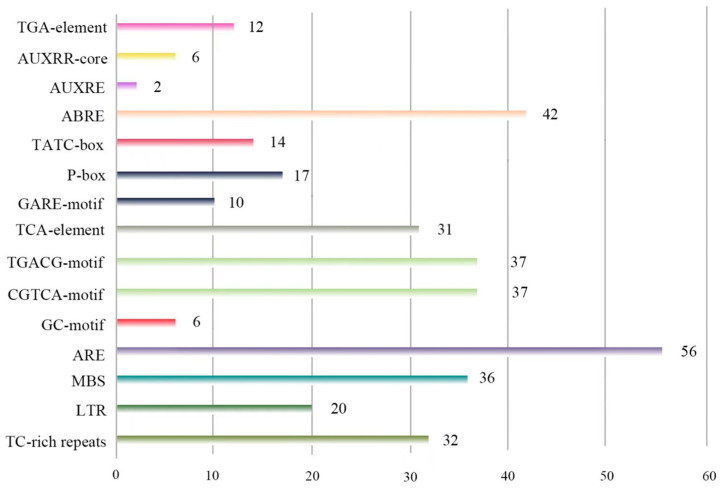
Numbers of *CsSAUR* genes containing different cis-acting elements.

The PlantCARE database was used to identify the cis-acting elements in the promoter region upstream of the transcriptional start site (−1 to −2000 bp) for all the identified *CsSAURs* in this study. Several such elements associated with environmental stress responses and phytohormone signaling were identified in these *CsSAUR* promoter regions (Figure 4 and Table S3), including the low-temperature-responsive (LTR) element, the TGA element, the AUXRE (auxin-responsive element) and the AUXRR core, the ABRE (ABA-responsive element), the TGACG motif and the CGTCA motif (MeJA-responsive elements), the GARE motif, the TATC box and P box (gibberellin-responsive) elements, and the TCA (salicylic acid-responsive element) elements. The ARE and ABRE elements were present in the promoters of 42 and 52 of these *CsSAUR* genes, respectively, with the most common elements being those related to responsivity to auxin, gibberellin, and MeJa. Multiple cis-acting elements were associated with tissue-specific patterns of expression including the CAT-box element and the GC motif, which are related to the expression in the meristem and endosperm, respectively.

### 2.5. Analyses of CsSAUR Expression Patterns

**Figure 5:**
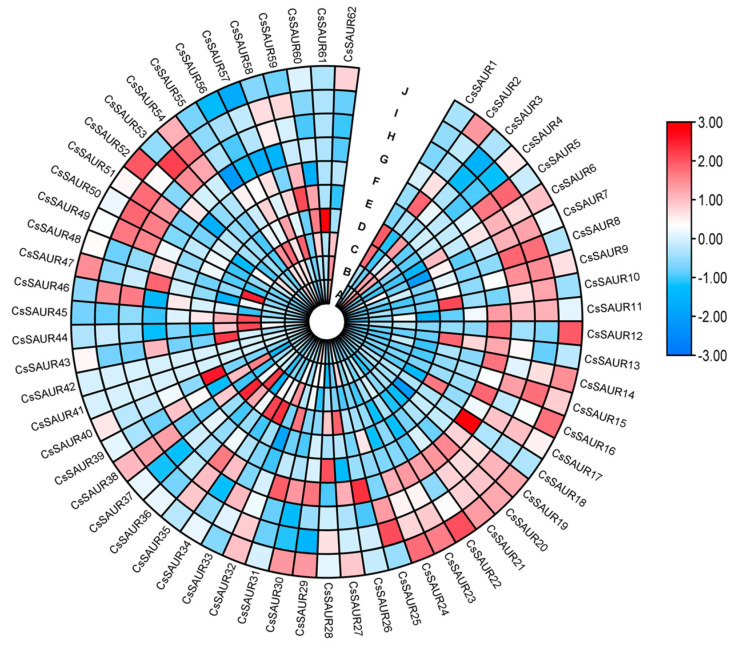
Heatmap highlighting the expression of different *CsSAUR* genes. *CsSAUR* genes in different cucumber organs: (**A**) ovary; (**B**) ovary (fertilized); (**C**) expanded ovary; (**D**) root; (**E**) stem; (**F**) leaf; (**G**) male flower; (**H**) tendril; (**I**) base part of tendril; (**J**) female flower tissues. The heatmap was constructed based on the FPKM. The colored bar indicates normalized gene expression in the log_2_ space.

To more fully explore the role that *CsSAUR* genes play in the development of cucumbers, the published RNA-seq data of 10 tissue in cucumber were used to generate a heat map to depict the tissue-specific expression of these SAUR proteins (Figure 5a). *CsSAUR41* and *CsSAUR42* were not detectable in any tissues, while all the other members of this family were highly expressed in at least one tissue. The majority of these *CsSAUR* genes were expressed in flowers, particularly male flowers, while *CsSAUR61* expression was only detectable in cucumber roots. Most *CsSAUR* genes were expressed in the ovary, root, stem, male flower, and tendril tissues of cucumber plants, wherein they may regulate development. The root tissues exhibited high levels of *CsSAUR27*, *CsSAUR31*, *CsSAUR43*, *CsSAUR47*, and *CsSAUR60* expression; high *CsSAUR40* levels were observed in the stem tissues; and high *CsSAUR8*, *CsSAUR46*, *CsSAUR49*, and *CsSAUR50* levels were evident in the tendrils, such that they may regulate elongation. *CsSAUR31* expression was found to be concentrated in the roots and male flowers, indicating that it may play a role in root and male flower development.

### 2.6. Phylogenetic and Sequence Alignment Analyses of CsSAUR31

**Figure 6:**
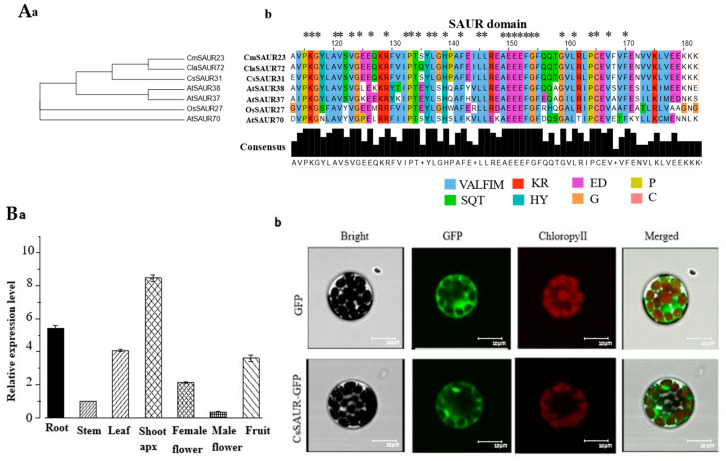
Sequence alignment, tissue expression, and subcellular localization analyses of CsSAUR31. (**A**) CsSAUR31 phylogenetic tree and sequence alignment. (**a**) Phylogenetic tree of CsSAUR31 and other SAUR proteins; (**b**) CsSAUR31 alignment with other SAUR proteins. (**B**) (**a**) *CsSAUR31* expression in different tissues; (**b**) CsSAUR31 subcellular localization. The amino acid residues at SAUR domain (about 60 amino acid residues) were labeled in different color. Asterisk (*) indicated highly conserved amino acid residues positions.

The full-length *CsSAUR31* cDNA sequence from the D0708 cucumber variety was cloned and amplified via RT-PCR, revealing a 489 bp coding region. The amino acid sequences encoded by *CsSAUR31* were obtained from cucumber plants, while the related SAUR protein sequences were gathered from other Cucurbitaceae species, and *SAUR37/38/70* sequences from *Arabidopsis* were also obtained. The resultant data revealed a close relationship between *CsSAUR31* and both *CmSAUR23* from melon (*Cucumis melo* L.) and *ClaSAUR72* from watermelon (*Citrullus lanatus* L.). The coding sequence for this gene contained a single auxin-inducible superfamily domain, consistent with its status as a SAUR family protein (Figure 6(Aa)). *CsSAUR31* may, thus, play functional roles similar to those of other members of the SAUR gene family. Conserved domain analyses indicated that CsSAUR31 shares a high degree of similarity with other SAUR proteins, with respect to their sequences (Figure 6(Ab)).

### 2.7. Expression and Subcellular Localization of CsSAUR31

To further gain insights into the functions of *CsSAUR31*, real-time quantitative polymerase chain reaction (RT-qPCR) analyses was carried out to measure the quantity of *CsSAUR31* transcripts in different organs. In D0708 plants, *CsSAUR31* levels were highest in the shoot apex and root tissues relative to male flowers, with moderate levels in female flowers, fruits, and leaves and only weak expression in the male flowers and stem tissues (Figure 6(Ba)). These results, thus, suggest that *CsSAUR31* may serve as an important regulator of root and shoot apex development.

Next, CsSAUR31 was subjected to subcellular localization analyses by constructing a construct encoding CsSAUR31−GFP protein under the control of the pSuper promoter. *Arabidopsis* protoplasts were then transfected with this CsSAUR31−GFP fusion expression vector or empty vector control. Subsequent confocal microscopy revealed that the GFP signal following *CsSAUR31−GFP* transfection was localized to the cytosol (Figure 6(Bb)), demonstrating that *CsSAUR31* encodes a cytoplasmic protein.

### 2.8. CsSAUR31 Functions as a Promoter of Hypocotyl and Root Elongation

**Figure 7:**
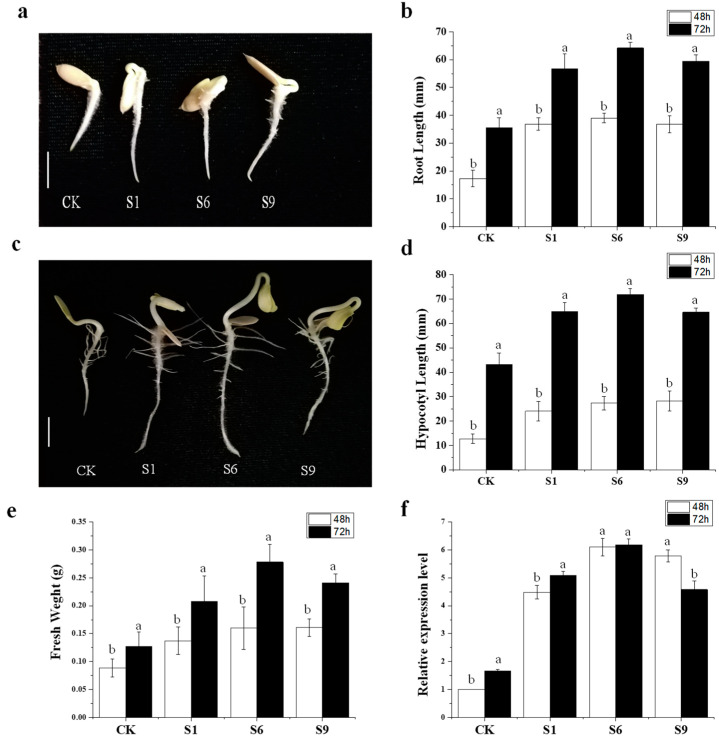
*CsSAUR31* regulates root and hypocotyl development in cucumber. (**a**) Root development of the control check (CK) and *CsSAUR31*-overexpressing (S) plants at 48 h. (**b**) Root length in CK and S at 48 h and 72 h. (**c**) Root development in CK and S at 72 h. (**d**) Hypocotyl lengths in CK and S at 48 h and 72 h. (**e**) Fresh weights in CK and S at 48 h and 72 h. (**f**) Relative root *CsSAUR31* expression levels in CK and S at 48 h and 72 h. The data are the means of three replicates with SEs, and different letters indicate a significant difference (*p* < 0.05 by Tukey’s test).

To directly examine the functional roles of *CsSAUR31* as a regulator of cucumber plant cell development, a CsSAUR31−PCXSN overexpression vector was constructed, with an empty vector under the control of the 35S promoter serving as a negative control. A RT-qPCR-based approach was used to identify 12 *CsSAUR31*-overexpressing plants and 1 control plant expressing the empty vector. Three of the overexpression lines expressing the highest *CsSAUR31* levels (lines 1, 6, and 9) were collected from the T1 generation following self-pollination (Figure S1). Relative to control plants, those overexpressing *CsSAUR31* exhibited enhanced growth (Figure 7a,c), with increases in root length (Figure 7b), hypocotyl length (Figure 7d), fresh weight (Figure 7e), and relative expression level of the roots (Figure 7f) at 48 and 72 h. These findings suggest that overexpressing *CsSAUR31* can promote enhanced plant cell growth and development.

### 2.9. Auxin Accumulation in the Context of CsSAUR31 Overexpression

**Figure 8:**
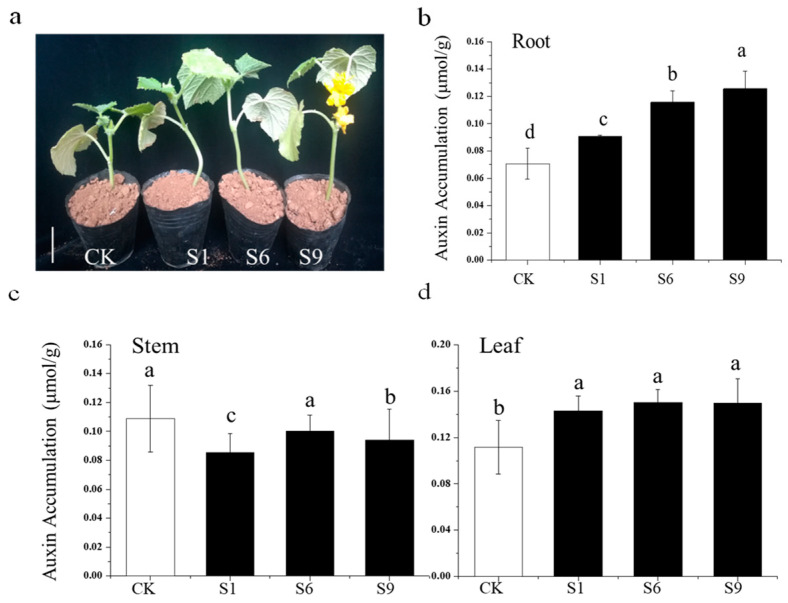
(**a**) Cucumber plant development of the control check (CK) and *CsSAUR31* overexpression (S). (**b**−**d**) Auxin levels in the roots (**b**), stems (**c**), and leaves (**d**) of the control check (CK) and *CsSAUR31*-overexpressing plants. The data are the means of three replicates with SEs, and different letters indicate a significant difference (*p* < 0.05 by Tukey’s test).

When they were first identified, *SAURs* were found to be present in auxin-treated elongated hypocotyls [28], indicating that they can be indicative of the elongation of auxin-dependent cells and organs [3]. *CsSAUR31* overexpression was able to promote root and hypocotyl growth in cucumber plants (Figure 7a,c and Figure 8a), much like the excess auxin production observed in the super root (*sur1* and *sur2*) mutants reported previously [29]. To directly assess the link between auxin and *CsSAUR31*-driven root elongation and other phenotypes, auxin concentrations were measured in the roots, stems, and leaves of cucumber seedlings. Relative to control plants, plants overexpressing *CsSAUR31* exhibited higher auxin levels in the root and leaf tissues (Figure 8b,d), although the auxin levels in the stem were reduced relative to those in control plants (Figure 8c).

## 3. Discussion

Here, 62 putative *CsSAUR* genes were identified through a bioinformatics analysis of the cucumber genome, and the protein sequences encoded by these genes were grouped into 7 groups, using an unrooted phylogenetic tree approach. *Arabidopsis SAUR* genes were closely related to *CsSAUR* genes in groups B, E, F, and G, and melon *SAUR* genes were also closely related to the same *CsSAURs*. The physical properties of the SAUR family members were also consistent across species, with molecular mass values in cucumber plants ranging from 9.474 (*CsSAUR47*) to 86.253 kDa (*CsSAUR37*), while in maize, tomato, and potato these molecular mass values range from 9.07 to 46.81 kDa [30], from 6.5 to 22.4 kDa, and from 9.1 to 25.1 kDa, respectively [6]. This highlights the high degree of evolutionary conservation for members of the *CsSAUR* gene family.

The 62 identified *CsSAUR* genes were unevenly distributed across all 7 cucumber chromosomes, with some being distributed in gene clusters, similar to what has been observed in *Arabidopsis*, maize, and tomato. For example, in *Arabidopsis*, eight, six, four, and six *SAUR* genes are clustered on chromosome 1 (*AtSAUR*61–68), chromosome 5 (*AtSAUR*19–24), chromosome 4 (*AtSAUR*1–5), and chromosome 4 (*AtSAUR*13–17), respectively [31]. Similar gene clusters are also identified in maize including *ZmSAUR8–12* (chromosome 1), *ZmSAUR*25–33 (chromosome 2), and *ZmSAUR*65–68 (chromosome 7) [11]. In tomato, 29 *SlSAUR* genes (*SlSAUR*3–31) are located in a cluster on chromosome 1, while chromosomes 4 and 11 harbor clusters of 10 (*SlSAUR*41–50) and 11 (*SlSAUR*87–97) members of this gene family, respectively, with an additional 16 being present on chromosome 10 [6]. Two *SAUR* gene clusters are also identified in the watermelon and melon genomes located on chromosomes 2/11 and 1/11, respectively [7,8]. Similarly, two *SAUR* gene clusters were, herein, identified in the cucumber genome on chromosomes 2 and 7, indicating a high degree of similarity for the *SAUR* genes across members of the Cucurbitaceae family. Eight motifs were identified within the CsSAUR proteins, with the majority of these proteins harboring motifs 1–3, such that they may be central to the ability of these proteins to regulate cucumber growth and development. Motif 4 was also present in 27 of these CsSAURs clustered on chromosomes 2 and 7, indicating a potentially distinct role for these CsSAURs as regulators of growth and development.

The majority of the *CsSAURs* and *SAURs* encoded by other species are associated with multiple hormone-responsive cis-acting elements including the TGA element, the AUXRR core, the AUXRE, the ABRE, the TATC box, the P box, the GARE motif, the TCA element, the TGACG motif, and the CGTCA motif elements. Plant hormones including auxin, ethylene, ABA, and salicylic acid (SA) are essential regulators of development and growth [32]. This indicates that these *SAURs* may serve as direct or indirect regulators of cucumber growth, with some being highly hormone-responsive and others being largely unresponsive. For example, *AtSAUR9*, *AtSAUR10*, *AtSAUR16*, *AtSAUR36*, *AtSAUR50*, and *ZmSAUR2* are generally auxin-responsive, whereas *AtSAUR8*, *AtSAUR12*, *AtSAUR19*, and *AtSAUR54* are not [9,10,11,12,13,14,15,18,33,34,35,36]. Auxin-inducible genes are more likely to exhibit promoter enrichment for the combined AuxRE element [33]. Even so, genes not inducible in response to auxin play an important role in controlling cellular elongation, given that *SAUR* overexpression can activate H+- ATPase activity in an auxin-independent manner [33,34]. Here, *CsSAUR31* was found to promote the elongation of cucumber hypocotyls and roots, despite lacking any known auxin-related *cis*-acting regulatory elements. *CsSAUR31* may, thus, engage similar regulatory mechanisms, with its overexpression obviating the need for auxin to promote elongation. However, more research is needed to study the mechanisms of action underlying *AtSAUR19* and *CsSAUR31*.

Some prior reports found SAUR fusion proteins to primarily exhibit nuclear localization [11]. However, these results are variable, with the *Arabidopsis* SAUR19 and SAUR63 proteins reportedly localized to the cell surface and plasma membrane, respectively [12,15], while OsSAUR39 and AtSAUR41 are localized to the cytosol [31,37]. Here, the protein encoded by CsSAUR31 was similarly found to be localized to the cytoplasm when expressed as a GFP fusion protein (Figure 6(Bb)). Despite the fact that CsSAUR31 and AtSAUR41 exhibit distinct patterns of localization compared to those of the proteins encoded by AtSAUR19 and AtSAUR63, *CsSAUR31* did promote similar phenotypes to those observed following the overexpression of *AtSAUR41*, *AtSAUR19*, and *AtSAUR63* subfamily genes [12,15,37]. For all of these previously studied genes, other than *OsSAUR39*, overexpression in seedlings is associated with hypocotyls that were elongated relative to wild-type seedlings [12,15,31]. An increase in vegetative biomass is evident following *AtSAUR41* and *AtSAUR19* overexpression [15,37], while primary root length and lateral root development are enhanced by *AtSAUR41* overexpression relative to wild-type plants. In this study, relative to control plants, primary root length and lateral root development were enhanced, and vegetative biomass was increased by CsSAUR31 overexpression (Figure 7a,c,e). These SAUR proteins may exhibit similar but nonidentical molecular functions and may potentially engage with different tissue-specific partner proteins to exert their specific effects. Other SAUR proteins exhibit opposing functions, as in the case of OsSAUR39 [31]. This may be attributable to the different molecular functions for different SAUR family protein subgroups or species-specific differences [31].

Auxin controls the expression of hundreds of different genes including members of the *SAUR* family [34,38]. Some *SAURs* are not responsive to auxin, owing to the lack of AuxRE elements, and these *SAURs* thus bypass the requirement for auxin to be expressed in the context of elongation growth [33,39,40]. Here, relative to levels in control plants, auxin levels were increased in the roots and leaves of cucumber plants overexpressing *CsSAUR31*, whereas they were lower in the stems of these plants (Figure 8b–d). *CsSAUR31* did not exhibit AuxRE or other auxin-responsive elements, and the mechanisms through which it can promote hypocotyl and root elongation may be similar to those reported previously for *AtSAUR19*, which is able to bypass normal auxin requirements to drive hypocotyl segment elongation [34]. Relative to control plants, the changes in tissue-specific auxin levels in those cucumber seedlings overexpressing *CsSAUR31* may be linked to the regulation of particular upstream or downstream genes. Additional studies are vital to fully clarify the functional roles of *CsSAUR31* in cucumber growth and to better understand how it interacts with auxin and other phytohormones.

## 4. Materials and Methods

### 4.1. SAUR Gene Identification and Phylogenetic Analysis

*Arabidopsis*, watermelon, and melon SAUR protein sequences were used as queries when searching the CuGenDBv2 database (http://cucurbitgenomics.org/v2/, accessed on 20 July 2022). All cucumber *SAUR* genes were identified via BLAST search (Cucumber Chinese long genome v3) [41]. Cucumber SAUR amino acid sequences, protein length, and molecular mass were predicted using ExPASy (http://web.expasy.org/protparam/, accessed on 20 July 2022) [42]. ClustalW was used for the multisequence alignment SAUR domains from cucumber, melon, and *Arabidopsis thaliana* [43], with results being imported into MEGA 11 for *SAUR* family rootless evolutionary tree construction [44]. The tree was constructed via a neighbor-joining (NJ) approach with 1000 bootstrap replicates, and p-distance was the model selection parameter. EvolView-v3 was used to edit the resultant tree (http://www.evolgenius.info/evolview/#/login, accessed on 20 July 2022) [45].

### 4.2. Chromosomal Location Analyses

CuGenDBv2 was used to identify the locations of all *CsSAUR* genes on individual chromosomes, after which TBtools software (https://github.com/CJChen/TBtools/v1.108/, accessed on 22 August 2022) was used to generate a location map [46]. Different *SAUR* groups were represented via coloration for clarity, with groups A–G being colored green, pink, blue, yellow, red, cyan, and orange, respectively.

### 4.3. Exon/Intron Structural Analyses and Conserved Motif Identification

TBtools software was used for conserved motif analyses of the *CsSAUR* family genes [46]. The maximum number of motifs was set to 20, and the amino acid width range was 6–50. Conserved domain details were gathered from the NCBI Conserved Domains Database (CDD) (https://www.ncbi.nlm.nih.gov/, accessed on 26 August 2022), while *SAUR* genes were obtained from Cucumber Chinese long genome v3 followed by online analysis using PlantCARE (http://bioinformatics.psb.ugent.be/webtools/plantcare/html/, accessed on 26 August 2022 ). The TBtools software analytical function was used to obtain the cucumber SAUR gene family exon and intron genetic structure patterns.

### 4.4. Cis-Acting Element Analyses

After designating the transcriptional start site as +1, promoter sequences for all SAUR genes spanning from −2 kb to +1 bp were obtained from Phytozome13, after which they were subjected to analysis with the PlantCARE online program to predict and locate cis-acting elements.

### 4.5. Analysis of the Expression Patterns of the Cucumber SAUR Genes Family

We obtained cucumber Illumina RNA-seq data from NCBI (PRJNA80169). Fragments per kilobase of exon model per million mapped reads (FPKM) values were used to represent the expression levels of SAUR genes. Differentially expressed genes (DEGs) were chosen based on the following criteria: *p*-value < 0.01, |log_2_ ratio ≥ 1|, and false discovery rate (FDR) < 0.05. We selected the transcriptome data of genes belonging to the SAUR genes family. The heat map was drawn, and then, the SAUR genes of cucumber tissue were analyzed. Expression levels were also analyzed. The specific tissues included ovary, expanded ovary, root, stem, leaf, male flower, tendril, base part of tendril, and female flower tissues TBtools software was used to perform the visualization of gene expression profile [46].

### 4.6. Plant Materials

Cucumber D0708 plants were obtained from the Cucumber Research Group of Northeast Agricultural University (Harbin, China). A total of 200 plants per genotype were grown in a solar greenhouse in pots filled with a mixture of soil and vermiculite (*v*/*v*: 1:1) at Northeast Agricultural University. Plants were cultivated under controlled conditions with a 75% relative humidity, a 12 h photoperiod, and 28 °C/18 °C (day/night).

### 4.7. CsSAUR31 Cloning and Bioinformatic Analysis

A full-length *CsSAUR31* coding sequence was obtained from the CuGenDBv2 database. Leaves from D0708 cucumber plants were used to clone the *CsSAUR31* gene using appropriate primers (Table S1), with PCR amplification using the following settings: 94 °C for 2 min; 30 cycles of 94 °C for 10 s, 56 °C for 15 s, and 72 °C for 10 s; and 72 °C for 5 min. The protein sequences for SAURs from other species were gathered from the NCBI database.

### 4.8. RT-qPCR

TRIzol (Invitrogen, Carlsbad, CA, USA) was used to extract RNAs from cucumber leaf samples, followed by its processing to produce first-strand cDNAs with a reverse transcription kit (Toyobo, Osaka, Japan). All RT-qPCR reactions were subsequently performed in a 20 µL volume that contained 2× Fast RT-qPCR Master Mix (DiNing, Beijing, China; 10 µL), cDNA (2 µL), forward and reverse primers (0.5 µL; 10 mM), and distilled water. Cucumber EF1a served as a normalization control when assessing relative gene expression [47] via the 2^−ΔΔCT^ method [48]. Thermocycler settings were as follows: 95 °C for 2 min; 40 cycles of 95 °C for 15 s, 56 °C for 15 s, and 72 °C for 30 s. A melt curve was generated using the default program on the qTower2.0 (Analytikjena, Jena, Germany). The RT-qPCR primers are listed in Table S1. Analyses were performed with three technical and three biological replicates.

### 4.9. Subcellular Localization Analyses

Full-length *CsSAUR31*, without the TAG codon, was amplified by PCR and cloned into a pSuper-1300 vector (containing GFP and a super promoter) through digestion (*Xba*I and *Bgl*II) and T4 ligation (Invitrogen, Carlsbad, CA, USA) [49]. An empty vector construct served as the negative control. *A. thaliana* protoplasts were then transfected with the control or CsSAUR31−GFP vectors and were subsequently imaged via confocal microscopy at 488 and 580 nm (Leica, Wetzlar, Germany) [50].

### 4.10. Cucumber Transformation

After amplification, *CsSAUR31* was cloned into a pCXSN-1250 vector, which contained a glyphosate resistance gene under the control of a constitutive promoter [51]. *Xcm*I was used to digest the construct, after which a TA cloning system was used, in accordance with the instructions of the manufacturer, and the *CsSAUR31* amplicons were ligated with T4 DNA ligase (Invitrogen, Carlsbad, CA, USA) to produce a pCXSN-1250-*CsSAUR31* overexpression vector. Cucumber plant cotyledons were infected with GV3101-derived agrobacteria to produce transgenic plants. Cells overexpressing *CsSAUR31* were selected using MS medium containing glufosinate (1 mg/L) [52]. T_0_ and T_1_ cucumber plants were validated through PCR and RT-qPCR using primers listed in Table S1.

### 4.11. Measurement of Auxin Levels

To assess auxin content in these plants, the root, stem, and leaf were collected from four leaf stages of the transgenic and control D07098 plants. Next, 0.1 g (±3%) samples of fresh plant tissue were suspended in PBS (pH 7.4) and fully homogenized, followed by centrifugation at 3000 rpm for 20 min. The supernatant was then collected, and auxin levels therein were measured via enzyme-linked immunosorbent assay kits (Meimian Industrial Co., Ltd., Yancheng, China). Differences in these levels were compared among groups using Student’s *t*-tests with *p* < 0.05 as the significance threshold.

## 5. Conclusions

In summary, in this study, a bioinformatics approach was used to identify the *SAUR* gene family members encoded by cucumber plants, after which these genes were subjected to classification, descriptive analyses, and further characterization. Overall, 62 *CsSAURs* distributed across all 7 chromosomes were identified and found to be expressed in a wide range of organs including flowers, leaves, stems, and roots, with some exhibiting tissue-specific patterns of expression. While *CsSAUR31* expression was detectable throughout the cucumber plant, its expression levels were highest in the roots and male flowers. CsSAUR31 was identified as a cytoplasmic protein that, when overexpressed, positively regulated cucumber root and hypocotyl development. These findings offer a robust basis for further efforts to functionally characterize cucumber SAUR proteins and will help support additional cucumber breeding efforts in the future.

## Data Availability

Not applicable.

## Supplementary Materials

The following supporting information can be downloaded at https://www.mdpi.com/article/10.3390/ijms24065940/s1.
