# Supplementary material for: Genome-Wide Identification and Functional Analysis of the Roles of SAUR Gene Family Members in the Promotion of Cucumber Root Expansion
Source: Int J Mol Sci. 2023 Mar 21;24(6):5940. doi: 10.3390/ijms24065940 (PMC10053606; doi:10.3390/ijms24065940)
Supplement: Supplementary file 1 [file ijms-24-05940-s001.zip › ijms-2239907-supplementary.pdf]

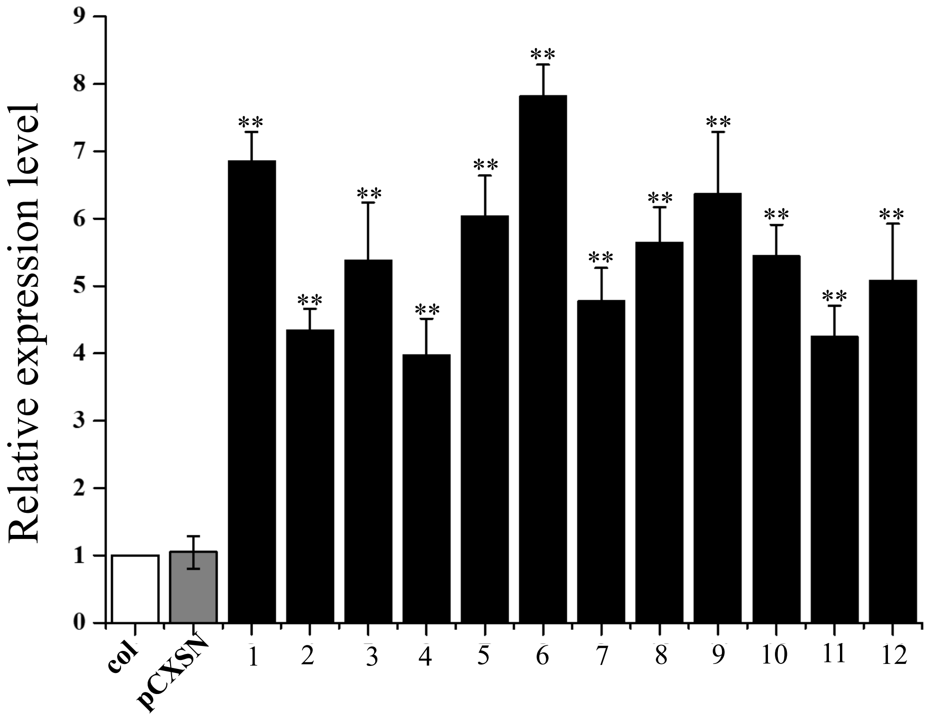

Figure S1: *CsSAUR31* levels in the control (Col), empty vector(pCXSN) and *CsSAUR31*-overexpressing (S) plants were screened by RT-qPCR. (**p < 0.01 by Student's t-test)

Table S1. Sequences of the oligo nucleotides used in this study

Oligo name	Oligo sequence（5′→ 3′）	Description	Gene ID
CsSAUR31-F	TTTGAGGTTGCCGTGTGAAG	RT-qPCR analysis	CsaV3_3G047300.1
CsSAUR31-R	GCACATTGGACTCTGTGGAC		
PCXSN-1250-F	ATGGATTCAAATGCAAAGAAGTCC	*CsSAUR31* overexpression detection	
PCXSN-1250-R	CTATCTGCACATTGGACTCTGTGG		

Table S2. Information of 62 *SAUR* genes, including the accession number, location (in which chromosome), length, molecular mass and pI

Gene name	Accession number	Chromosome	Length (a.a.)	Molecular weight (kDa)	pI	Instability Index	Grand Average of Hydropathicity
CsSAUR1	CsaV3_1G009830.1	1	158	17199.65	8.67	42.07	-0.304

CsSAUR2	CsaV3_1G013410.1	1	143	16173.61	6.91	40.69	-0.464
CsSAUR3	CsaV3_2G015370.1	2	153	17254.82	9.66	67.52	-0.409
CsSAUR4	CsaV3_2G015380.1	2	103	11617.36	6.4	61.59	-0.284
CsSAUR5	CsaV3_2G015400.1	2	97	10959.63	8.69	21.94	-0.17
CsSAUR6	CsaV3_2G015410.1	2	97	10937.86	9.58	39.83	-0.003
CsSAUR7	CsaV3_2G015420.1	2	95	10598.18	7.77	29.35	-0.007
CsSAUR8	CsaV3_2G015430.1	2	95	10616.14	7.87	59.19	-0.168
CsSAUR9	CsaV3_2G015450.1	2	95	10904.59	9.43	48.53	-0.266
CsSAUR10	CsaV3_2G015460.1	2	97	10939.7	9.14	54.31	-0.112
CsSAUR11	CsaV3_2G015480.1	2	97	10716.42	9.25	63.88	-0.074
CsSAUR12	CsaV3_2G015490.1	2	97	10859.66	9.03	46.44	-0.138
CsSAUR13	CsaV3_2G015500.1	2	132	14787.96	9.3	55.71	-0.124
CsSAUR14	CsaV3_2G015510.1	2	97	10939.66	8.89	42.31	-0.134
CsSAUR15	CsaV3_2G015520.1	2	97	10897.68	9.39	51.55	-0.099
CsSAUR16	CsaV3_2G015530.1	2	97	10884.73	9.03	44.59	-0.085
CsSAUR17	CsaV3_2G015540.1	2	97	10983.78	8.85	40.95	-0.057
CsSAUR18	CsaV3_2G015550.1	2	97	11127.07	9.37	43.93	-0.032
CsSAUR19	CsaV3_2G015560.1	2	97	10838.66	9.62	51.49	-0.021
CsSAUR20	CsaV3_2G015570.1	2	97	10886.78	8.79	45.01	0.054
CsSAUR21	CsaV3_2G015580.1	2	92	10518.37	9.51	43.77	-0.116
CsSAUR22	CsaV3_2G015590.1	2	205	23406.58	9.33	50.84	-0.061
CsSAUR23	CsaV3_2G015600.1	2	97	10957.82	9.44	61.32	-0.079
CsSAUR24	CsaV3_2G015610.1	2	97	11034.93	9.69	52.49	-0.086
CsSAUR25	CsaV3_2G029400.1	2	115	13047.06	9.15	58.24	-0.309
CsSAUR26	CsaV3_3G003060.1	3	153	17745.7	10.38	33.62	-0.436
CsSAUR27	CsaV3_3G007730.1	3	109	12881.54	8.96	62.43	-0.962
CsSAUR28	CsaV3_3G030900.1	3	101	11334.93	6.02	48.94	-0.091
CsSAUR29	CsaV3_3G045710.1	3	200	22858.37	9.58	73.52	-0.374
CsSAUR30	CsaV3_3G045730.1	3	100	11587.62	10.29	66.57	-0.389
CsSAUR31	CsaV3_3G047300.1	3	141	15548.04	9.57	41.64	-0.32
CsSAUR32	CsaV3_4G031250.1	4	113	13701.69	8.54	65.14	-0.864
CsSAUR33	CsaV3_4G031860.1	4	150	17398.53	4.85	58.2	-0.649
CsSAUR34	CsaV3_5G000180.1	5	121	14261.21	7.05	34.71	-0.464
CsSAUR35	CsaV3_5G027650.1	5	167	18882.37	5.45	52.28	-0.585
CsSAUR36	CsaV3_6G001030.1	6	176	20852.25	10.28	59.76	-0.557
CsSAUR37	CsaV3_6G007970.1	6	746	86253.06	6.4	49.54	-0.498
CsSAUR38	CsaV3_6G008790.1	6	159	18513.11	9.54	47.57	-0.784
CsSAUR39	CsaV3_6G013600.1	6	159	18108.83	9.63	58.09	-0.306
CsSAUR40	CsaV3_6G014200.1	6	154	17186.25	4.77	58	-0.505
CsSAUR41	CsaV3_6G036570.1	6	162	18887.15	8.96	42.77	-0.291
CsSAUR42	CsaV3_6G039880.1	6	143	16650.2	5.34	56.41	-0.212
CsSAUR43	CsaV3_6G040760.1	6	340	17175.71	6.91	45.47	-0.227

CsSAUR44	CsaV3_6G047000.1	6	385	12193.05	8.93	57.42	-0.301
CsSAUR45	CsaV3_7G000560.1	7	374	17111.59	10.04	80.53	-0.399
CsSAUR46	CsaV3_7G000570.1	7	486	10289.99	8.8	54.22	-0.266
CsSAUR47	CsaV3_7G000580.1	7	352	9473.87	7.83	43.71	-0.248
CsSAUR48	CsaV3_7G000590.1	7	212	10663.13	6.71	59.9	-0.148
CsSAUR49	CsaV3_7G000600.1	7	274	11224.57	5.04	58.63	-0.532
CsSAUR50	CsaV3_7G000620.1	7	239	12799.42	5.73	54.01	-0.413
CsSAUR51	CsaV3_7G000630.1	7	213	10943.42	6.72	55.57	-0.305
CsSAUR52	CsaV3_7G000650.1	7	299	11158.69	6.06	52.5	-0.225
CsSAUR53	CsaV3_7G000660.1	7	262	10194.93	8.91	44.69	-0.043
CsSAUR54	CsaV3_7G000680.1	7	140	18438.28	9.04	37.57	-0.23
CsSAUR55	CsaV3_7G000690.1	7	196	79309.37	4.86	44.78	-0.778
CsSAUR56	CsaV3_7G000700.1	7	461	19409.84	9.36	51.58	-0.024
CsSAUR57	CsaV3_7G000710.1	7	295	11306	7.81	23.48	-0.068
CsSAUR58	CsaV3_7G000720.1	7	327	12819.65	6.8349	39.54	-0.281
CsSAUR59	CsaV3_7G001480.1	7	168	17460.54	9.7743	60.99	0.013
CsSAUR60	CsaV3_7G022810.1	7	153	14460.62	9.4923	87.94	-0.192
CsSAUR61	CsaV3_7G033620.1	7	155	18153.91	10.2848	55.04	-0.494
CsSAUR62	CsaV3_7G034130.1	7	327	13791.74	6.8349	46.98	-0.011

Table S3. Information for some *cis*-elements

Gene name	TGA-element	AuxRR-core	AUXRE	ABRE	TATC-box	P-box	GARE-motif	TCA-element	TGACG-motif	CGTCA-motif	GC-motif	ARE	MBS	LTR	TC-rich repeats	sum
CsSAUR1				1			1	1				1		1	1	6
CsSAUR2				1				1				1			1	4
CsSAUR3	1			1				1	1	1		1	1			7
CsSAUR4	1			1			1	1				1			1	6

CsSA UR5	1			1							1	1	1		1	6
CsSA UR6	1			1							1	1	1		1	6
CsSA UR7	1			1	1			1				1	1	1		7
CsSA UR8								1				1	1			3
CsSA UR9				1	1				1	1		1	1			6
CsSA UR10		1				1			1	1		1	1	1	1	8
CsSA UR11				1		1					1	1	1			5
CsSA UR12				1								1	1		1	4
CsSA UR13						1			1	1		1	1		1	6
CsSA UR14		1						1	1	1		1	1		1	7
CsSA UR15			1		1							1	1		1	5
CsSA UR16				1					1	1		1	1	1		6
CsSA UR17			1	1					1	1		1	1	1	1	8
CsSA UR18				1					1	1		1	1		1	6
CsSA UR19	1			1		1		1	1	1		1	1			7

CsSA UR20				1		1		1	1	1		1	1			7
CsSA UR21	1					1	1	1	1	1		1	1			8
CsSA UR22					1		1					1	1		1	5
CsSA UR23					1			1				1	1	1	1	6
CsSA UR24								1					1		1	3
CsSA UR25				1	1			1	1	1	1	1	1			8
CsSA UR26				1	1	1		1				1	1			6
CsSA UR27	1			1	1			1				1				5
CsSA UR28				1				1				1	1		1	5
CsSA UR29				1				1	1	1	1	1		1	1	8
CsSA UR30						1						1				2
CsSA UR31				1			1	1				1		1		5
CsSA UR32				1				1	1	1		1			1	6
CsSA UR33				1	1				1	1		1				5
CsSA UR34				1		1			1	1				1	1	6

CsSA UR35				1			1		1	1		1	1	1	1	8
CsSA UR36				1		1		1	1	1						5
CsSA UR37				1								1	1			3
CsSA UR38				1				1	1	1		1		1		6
CsSA UR39				1		1			1	1			1	1		6
CsSA UR40						1		1	1	1			1		1	6
CsSA UR41	1			1			1		1	1		1		1		7
CsSA UR42				1		1		1	1	1		1				6
CsSA UR43	1								1	1		1				3
CsSA UR44				1	1		1		1	1			1	1	1	8
CsSA UR45				1					1	1		1			1	5
CsSA UR46		1						1	1	1		1		1	1	7
CsSA UR47					1	1		1				1	1		1	6
CsSA UR48				1					1	1		1		1	1	6
CsSA UR49		1		1					1	1		1	1	1		7

CsSA UR50									1	1		1	1			4
CsSA UR51									1	1		1				3
CsSA UR52				1	1	1	1	1	1	1		1	1		1	10
CsSA UR53							1					1	1	1	1	5
CsSA UR54									1	1	1	1			1	5
CsSA UR55				1					1	1		1	1		1	6
CsSA UR56				1				1	1	1		1			1	6
CsSA UR57				1		1			1	1		1		1		6
CsSA UR58		1		1		1		1				1		1		6
CsSA UR59	1	1			1							1			1	6
CsSA UR60								1				1	1			3
CsSA UR61				1				1				1				3
CsSA UR62	1			1				1	1	1		1				6
